# Exacerbated central fatigue and reduced exercise capacity in early-stage breast cancer patients treated with chemotherapy

**DOI:** 10.1007/s00421-023-05177-5

**Published:** 2023-03-20

**Authors:** Elyse Hucteau, Joris Mallard, Xavier Pivot, Roland Schott, Carole Pflumio, Philippe Trensz, Fabrice Favret, Allan F. Pagano, Thomas J. Hureau

**Affiliations:** 1 Biomedicine Research Centre of Strasbourg (CRBS), Mitochondria, Oxidative Stress, and Muscular Protection Laboratory (UR 3072), Strasbourg, France; 2 Faculty of Sport Sciences, European Centre for Education, Research and Innovation in Exercise Physiology (CEERIPE), University of Strasbourg, 4 rue Blaise Pascal, CS 90032, 67081 Strasbourg Cedex, France; 3 Institute of Cancerology Strasbourg Europe (ICANS), Strasbourg, France

**Keywords:** Neuromuscular fatigue, Central and peripheral fatigue, Neuromuscular function, Exercise tolerance, Critical force

## Abstract

**Purpose:**

The present study aimed to characterize the etiology of exercise-induced neuromuscular fatigue and its consequences on the force-duration relationship to provide mechanistic insights into the reduced exercise capacity characterizing early-stage breast cancer patients.

**Methods:**

Fifteen early-stage breast cancer patients and fifteen healthy women performed 60 maximal voluntary isometric quadriceps contractions (MVCs, 3 s of contraction, 2 s of relaxation). The critical force was determined as the mean force of the last six contractions, while *W*’ was calculated as the force impulse generated above the critical force. Quadriceps muscle activation during exercise was estimated from vastus lateralis, vastus medialis and rectus femoris EMG. Central and peripheral fatigue were quantified via changes in pre- to postexercise quadriceps voluntary activation (ΔVA) and quadriceps twitch force (ΔQTw) evoked by supramaximal electrical stimulation, respectively.

**Results:**

Early-stage breast cancer patients demonstrated lower MVC than controls preexercise (− 15%, *P* = 0.022), and this reduction persisted throughout the 60-MVC exercise (− 21%, *P* = 0.002). The absolute critical force was lower in patients than in controls (144 ± 29N vs. 201 ± 47N, respectively, *P* < 0.001), while *W*’ was similar (*P* = 0.546), resulting in lower total work done (− 23%, *P* = 0.001). This was associated with lower muscle activation in the vastus lateralis (*P* < 0.001), vastus medialis (*P* = 0.003) and rectus femoris (*P* = 0.003) in patients. Immediately following exercise, ΔVA showed a greater reduction in patients compared to controls (− 21.6 ± 13.3% vs. − 12.6 ± 7.7%, *P* = 0.040), while ΔQTw was similar (− 60.2 ± 13.2% vs. − 52.8 ± 19.4%, *P* = 0.196).

**Conclusion:**

These findings support central fatigue as a primary cause of the reduction in exercise capacity characterizing early-stage breast cancer patients treated with chemotherapy.

**Clinical trials registration:**

No. NCT04639609—November 20, 2020.

## Introduction

Cancer-related fatigue is defined as a subjective sense of tiredness related to cancer or its treatment that interferes with normal functions (Mock 2001), and is assessed using self-report questionnaires (Al Maqbali et al. 2019; Jacobsen 2004). In breast cancer, it has been documented that cancer-related fatigue reached a 75% prevalence following the first cycle of chemotherapy (Peoples et al. 2017), increased significantly during the first three months of adjuvant chemotherapy (Binotto et al. 2020), and can persist for years after treatment completion (Goldstein et al. 2012). Importantly, cancer-related fatigue is directly associated with an impairment of the quality of life in patients with breast cancer (Binotto et al. 2020; Bower et al. 2000). While the 5-year survival rate is currently 87% in patients with breast cancer (Allemani et al. 2018), understanding and alleviating cancer-related fatigue is of particular interest to improve the quality of life of these patients.

Identified as a multifactorial symptom, cancer-related fatigue is determined by biological, demographic, psychosocial, behavioral and physiological factors (Al-Majid and Gray 2009; Bower 2014). Among physiological factors, alterations of the neuromuscular function have been recently identified as a predictor of cancer-related fatigue in patients with breast cancer (Veni et al. 2019; Chartogne et al. 2021; Brownstein et al. 2021). Specifically, these alterations driven by the disease and the treatments include a decrease in muscle mass (Mallard et al. 2022a; Guigni et al. 2018a), a decrease in muscle force (Mallard et al. 2022b; Mijwel et al. 2018; Klassen et al. 2017), and an increase in neuromuscular fatigue (Klassen et al. 2017). Neuromuscular fatigue is quantified by the transient reduction of the muscle’s ability to generate a force (Enoka and Duchateau 2008) that can be related to a failure of the central nervous system to voluntarily activate the muscle (i.e., central fatigue) (Gandevia 2001) and/or to biochemical changes within the active muscle leading to an attenuated response to neural output (i.e., peripheral fatigue) (Bigland-Ritchie et al. 1978).

While previous studies consensually identified exacerbated neuromuscular fatigue in various cancer survivors several months or years post-treatment, the mechanisms involved were equivocal with studies showing greater fatigue from the either central or peripheral origin (Cai et al. 2014; Kisiel-Sajewicz et al. 2012, 2013; Yavuzsen et al. 2009; Brownstein et al. 2022; Neil et al. 2013; Prinsen et al. 2015; Lavigne et al. 2020). However, if studies performed on these cancer survivors are insightful in prescribing exercise or other interventions to improve their quality of life after treatment, it is also crucial to characterize patients’ maladaptations to prescribe such interventions during treatment. Moreover, it is important to specifically target a cancer type, as cancers have very different symptoms, treatment options, and outcomes (Lin 2019; Hickok et al. 2005; Baracos et al. 2018). Indeed, it has been evidenced that neuromuscular alterations differed between cancer types (Christensen et al. 2014). To date, there is no study investigating the net effect of cancer treatment on neuromuscular fatigue immediately at treatment completion (i.e., without substantial recovery time such as studies in cancer survivors), in patients with breast cancer.

Cancer-related fatigue has also been associated with a reduction in exercise capacity (Neil et al. 2013; Jung et al. 2019). In this context, key parameters derived from the force–duration relationship, namely the critical force and the curvature constant (*W*’), provide a cohesive framework within which to investigate the mechanistic bases of neuromuscular fatigue and exercise capacity (Poole et al. 2016). Mathematically, critical force is defined as the asymptote of the hyperbolic force-duration relationship, whereas *W*’ represents the fixed amount of force impulse that can be performed above critical force (Burnley 2009). Physiologically, critical force is associated with the greatest oxidative metabolic rate that can be sustained without a continuous reduction in *W*’ (Poole et al. 2016). The magnitude of *W*’ depletion is associated with the development of neuromuscular fatigue (Poole et al. 2016; Zarzissi et al. 2020) and the loss of muscular efficiency (Murgatroyd and Wylde 2011). Therefore, the characterization of critical force and *W*’ would provide additional bioenergetic insights underpinning both neuromuscular fatigue and exercise capacity in patients with breast cancer. Of note, these parameters have only been investigated during handgrip exercise in cancer patients/survivors (Veni et al. 2019; Chartogne et al. 2021) but not exercising locomotor muscles such as the quadriceps, which would be more relevant to locomotion and activities of daily living (Winters-Stone et al. 2008).

Therefore, the present study aimed to characterize the etiology of exercise-induced quadriceps neuromuscular fatigue and to determine the parameters of the force-duration relationship in early-stage breast cancer patients at chemotherapy completion compared to well-matched healthy controls. It was hypothesized that patients would develop exacerbated neuromuscular fatigue compared to their healthy counterparts due to elevated cancer-related fatigue. Consequently, exercise capacity (i.e., total work done) was expected to be lower in patients than in controls and characterized by a reduction in absolute critical force and an increase in *W*’.

## Methods

### Participants

Fifteen patients with breast cancer were included in the study (patient group). Eligibility criteria included French-speaking, nonpregnant, ≥ 18 years old, women with Scarff‐Bloom‐Richardson grade I–III and early-stage breast cancer (1–3), World Health Organization (WHO) performance status between 0 and 2, within 2 weeks after completion of anthracycline-cyclophosphamide and taxane-based (neo)adjuvant chemotherapy.

Women were excluded if they had psychiatric, musculoskeletal, or neurological disorders.

Fifteen healthy volunteer women (control group) were matched to the characteristics of the breast cancer group regarding age, weight, height, and physical activity level. Eligibility and exclusion criteria were the same as those identified in the patient group except for criteria related to breast cancer status. Indeed, participants from the control group had no history of cancer or another chronic disease.

The characteristics of the participants included in the present study (NCT04639609) are presented in Table 1. All participants provided written informed consent before enrollment, and the study was conducted in accordance with both the Declaration of Helsinki and the ethics approval received from the national ethics committee (2020-A01272-37).

**Table 1:** Participants’ characteristics

	Controls (*n* = 15)	Patients (*n* = 15)	*P* value
Matched characteristics (mean ± SD)			
Age, year	47 ± 8	47 ± 9	0.948
Weight, kg	73 ± 14	72 ± 13	0.839
Height, cm	167 ± 5	165 ± 6	0.399
Physical activity level, MET-min/week	1108 ± 530	1191 ± 504	0.573
Body composition (mean ± SD)			
Body mass index, kg/m^2^	26 ± 5	26 ± 4	0.830
Fat-free mass, kg	45 ± 9	45 ± 6	0.539
Fat mass, kg	26 ± 9	27 ± 9	0.838
Skeletal muscle mass, kg	21 ± 3	20 ± 4	0.262
Cancer characteristics			
Tumor stage (*n*)			
2	–	9	–
3	–	6	–
Tumor SBR grade (*n*)			
I	–	1	–
II	–	9	–
III	–	5	–
Tumor type (*n*)			
Triple negative	–	7	–
Luminal (A/B)	–	7	–
HER2-positive	–	1	–
Treatment setting (*n*)			
Adjuvant	–	6	–
Neo-adjuvant	–	9	–
Neuromuscular function parameters (mean ± SD)			
MVC, *N*	368 ± 89	303 ± 54	0.022
VA, %	94 ± 5	94 ± 4	0.589
Q_tw_, *N*	139 ± 39	132 ± 21	0.523

*HER2* human epidermal growth factor receptor 2, *MVC* maximal voluntary contraction, *Q*_*tw*_ quadriceps twitch amplitude, *SBR* Scarff‐Bloom‐Richardson grade, *VA* voluntary activation

### Experimental protocol

**Fig. 1:**
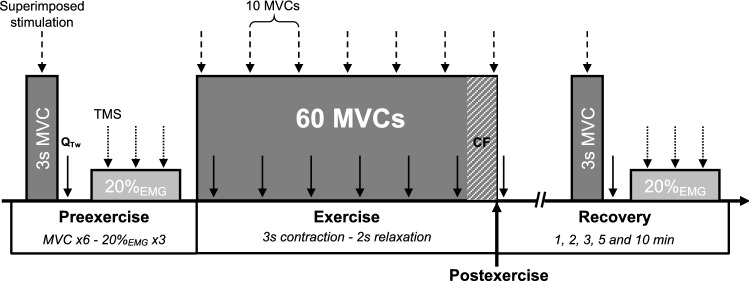
Schematic illustration of the experimental protocol. Neuromuscular assessments were performed on knee extensors. Electrical femoral nerve stimulations were delivered during (dashed arrow) and after maximal voluntary contractions (MVCs) (solid arrow, Q_tw_). Transcranial magnetic stimulations (TMS) were delivered during a 20% EMG-RMS submaximal contraction (dotted arrow) to quantify corticospinal excitability. *CF* critical force

All participants carried out the experimental protocol, conducted as follows: (1) familiarization with isometric knee extensor contractions and the associated experimental procedures, followed 30 min later by (2) neuromuscular function assessment (i.e., preexercise), (3) the fatiguing exercise task, (4) neuromuscular function assessment throughout 10 min of recovery (i.e., postexercise) (Fig. 1).

Neuromuscular function of the knee extensors was investigated with femoral electrical nerve and transcranial magnetic stimulations. After a standardized warm-up including submaximal isometric knee extensor contractions, participants were asked to perform six 3 s-MVCs, separated by 30 s recovery to ensure full potentiation. Further attempts were made if the variability in MVC exceeded 5%. Stimulations were applied both during (superimposed twitch) and 1 s after (potentiated resting twitch, Q_tw_) each MVC to assess voluntary quadriceps activation and muscle contractile properties, respectively (Nuzzo et al. 2018). For all attempts, strong verbal encouragement was given. Corticospinal excitability was measured during submaximal voluntary isometric contractions corresponding to 20% of each participant’s vastus lateralis maximal EMG root mean square (EMG-RMS) output obtained from MVC. We used submaximal contractions based on a constant EMG activity to counteract the confounding increase in corticospinal excitability associated with an increase in EMG during a fatiguing constant force exercise (Lévénez et al. 2008; Hoffman et al. 2009). These submaximal contractions corresponded to 26 ± 5% of the MVC force. Using visual feedback, participants were asked to perform three submaximal contractions for 5–10 s and separated by 30 s of recovery (McNeil et al. 2011). During each contraction, 3 transcranial magnetic stimulations were delivered.

Participants were then required to perform a fatiguing task consisting of 60 MVCs over a 5 min period (3 s contraction, 2 s relaxation) with strong verbal encouragement. A target line was set on the computer screen at 100% MVC to maximize performance, and an audio recording cued the start and stop of each MVC to follow the required duty cycle. Superimposed and potentiated resting electrical stimulations were elicited at first and every ten contractions to investigate neuromuscular fatigue development throughout the exercise. No magnetic stimulation was applied during the 60-MVC protocol.

After the fatiguing task, neuromuscular function was assessed immediately and at 1, 2, 3, 5, and 10 min to quantify recovery. MVC was performed with superimposed and potentiated resting twitches for each set, followed by a 20% EMG-RMS submaximal contraction with 3 superimposed transcranial magnetic stimulations.

### Experimental procedures

#### Force and electromyogram recording

Participants were tested in a seated position with the hip and knee joints fixed, respectively, at 100° and 90° (where 180° represents a full extension) and aligned in the frontal axis. The lower right leg was strapped above the ankle to an ergometer connected to a force transducer. The quadriceps force of the right leg obtained from both evoked and voluntary contractions was collected using a calibrated force transducer (Force sensor kit, Chronojump, Barcelona, Spain). Surface electromyogram (EMG) recording electrodes (Ag–AgCl, 32 × 32 mm) were placed bilaterally over the vastus lateralis (VL), vastus medialis (VM), rectus femoris (RF), and biceps femoris (BF) according to the SENIAM-recommended guidelines (Hermens et al. 2000). EMG recordings were bandpass filtered (1 kHz–10 Hz) and amplified with an isolated differential amplifier (Octal Bio Amp, AD Instruments, Bella Vista, New South Wales, Australia). Electromyographic and mechanical signals were collected simultaneously at a sampling rate of 2000 Hz in LabChart Software (AD Instruments, Bella Vista, New South Wales, Australia, Version 8). During the 60-MVC protocol, VL, VM, and RF muscle activation was calculated with RMS on EMG signals. RMS was determined for each contraction over a 500-ms interval corresponding to the peak force.

#### Electrical femoral nerve stimulation

A high-voltage (400 V) Digitimer stimulator (Model DS7-AH, Digitimer Ltd., Hertfordshire, UK) was used to generate single electrical stimulations (rectangular pulses of 200 µs duration). Electrical stimulations were applied to the femoral nerve to evoke quadriceps contractions. The cathode (32 mm diameter, Valu Trode, CF3200 models, Denmark) was positioned over the inguinal space on the femoral nerve and the anode (90 × 50 mm, Valu Trode, 895240 models, Denmark) in the gluteal fold. A recruitment curve was performed to determine the optimum stimulation intensity with increasing intensity at 10 mA initiated from 50 mA until maximal single twitch peak force (Q_tw_) and compound muscle action potential (M-wave) were achieved. The maximal intensity was reached when no increase in Q_tw_ and M-wave (M_max_) was observed between the two stimulation intensities. A supramaximal stimulation intensity (i.e., 120% of the maximal stimulation intensity) was used to guarantee the complete recruitment of motor units.

#### Transcranial magnetic stimulation

A transcranial magnetic stimulator (Magstim 200^2^, The Magstim Company Ltd, Dyfed, UK) was used to generate magnetic stimulation via a double-cone coil (diameter of 130 mm). Intensities are expressed as a percentage and function of the maximum output intensity (1.4T). Stimulations were applied to the left motor cortex to evoke motor-evoked potentials (MEPs) in the right quadriceps with the current flowing posterior to anterior direction. Coil position was determined by applying stimulations at 50% during 20% EMG-RMS isometric contraction registered from the quadriceps MVC. The optimal position, where the greatest possible MEP in the VL and the lowest in the BF (< 15% of raw VL MEP amplitude), was marked directly on the scalp with a medical skin marker for reproducibility during the experimental session. Then, the stimulation intensity was determined by the active motor threshold. The motor threshold was the minimum stimulus intensity required to elicit an MEP (more than 50 µV in 50% of trials) during 20% EMG-RMS isometric contraction. The stimulator intensity was set to 120% of the active motor threshold to ensure a clear MEP (Gruet et al. 2013).

### Complementary assessments

#### Muscle architecture

The muscle architecture of the VL was investigated before the neuromuscular investigations using an ultrasound scanner (Toshiba Aplio XV; Toshiba Medical Systems, Tochigi, Japan) with a 58 mm linear probe (PLT-805AT 8.0-MHz). The participant was lying down with the knee in the extended position and muscles relaxed. A water-soluble transmission gel was applied to the scanning head of the probe. The probe was placed perpendicular and longitudinally to the skin at 62.5% of the distance between the anterior superior iliac spine and the lateral condyle of the femur. Five ultrasound images were taken and stored. Images were then analyzed using ImageJ (version 1.8.0, National Institutes of Health, Bethesda, MD, USA). Muscle thickness, pennation angle, and fascicle length were determined using a validated methodology (Ando et al. 2014). For each variable, five different images were analyzed by two independent investigators, and these data were averaged to ensure reliability.

#### Body composition

Body mass, body mass index, fat-free mass, fat mass, and skeletal muscle mass were quantified using a validated bioelectrical impedance meter (SECA mBCA 515, SECA, Hamburg, Germany) (Bosy-Westphal et al. 2013).

#### Physical activity level, quality of life and cancer-related fatigue

Physical activity level was assessed using the GPAQ questionnaire version 2 (Armstrong and Bull 2006). Quality of life was self-assessed using the FACT-G in patients and the FACT-GP in controls (items related to illness or treatment were removed for healthy participants) (Butt et al. 2013). Cancer-related fatigue was specifically assessed using the FACIT-F subscale.

### Data analysis

The critical force during the 60-MVC protocol was the mean force recorded over the last six MVCs, while *W*’ was calculated as the total force impulse generated above the critical force (Burnley 2009). The total work done was also determined and calculated as the total force impulse generated during the 60-MVC protocol. Q_tw_ was calculated as the force amplitude between the baseline signal and the highest force value of the evoked potentiated twitch. Moreover, the following parameters of potentiated resting twitch, as indicators of muscle contractile properties, were also calculated: contraction time (CT), which is the time from the start of the contraction to Q_tw_; half relaxation time (HRT), which is the time from Q_tw_ to 50% decline in Q_tw_; maximal rate of force development (RFD), which is the steepest rate of torque development (i.e., highest positive derivative of the torque for an interval of 10 ms between two cursors placed on either side of the torque rise). Voluntary activation was calculated using the twitch interpolation method (Merton 1954), with the amplitude of the superimposed twitch (SIT) and Q_tw_, using the following formula: Voluntary activation (%) = [1 − (SIT ÷ Q_tw_) $\times$ 100]. The peak-to-peak amplitudes of M_max_ and MEP were measured between maximum and minimum values for the electromyographic signals. To account for any alteration of muscle sarcolemma excitability and variability associated with EMG measurements (Lepers et al. 1985), the amplitude of each MEP was normalized to concomitant M_max_ (MEP.cM_max_^−1^), and RMS data were then normalized to the root mean square recorded during pre-exercise MVCs (RMS_%MVC_)_._ The 3 MEP.cM_max_^−1^ obtained during each submaximal contraction were averaged together to minimize the variability of this parameter.

### Statistical analysis

The sample size calculation was based on a previous investigation documenting MVC in breast cancer patients at the end of the adjuvant treatment compared to healthy controls (Gomes et al. 2014). Assuming an effect size of 0.96, $\alpha$ = 0.05, and $\beta$ = 0.8, the minimum number of participants required to establish a significant difference in maximal voluntary force between groups was calculated at fifteen per group (G*power, version 3.1.9.4).

All statistical tests and graphs were generated with GraphPad Prism version 8 software (GraphPad Software, San Diego, California, USA). Data are presented as the means ± SD. The Shapiro–Wilk test and the Levene test were used to check for normality and variance homogeneity of the data, respectively. Paired-samples t-tests were used to compare participants’ characteristics (age, height, body mass, body mass index, fat-free mass, fat mass, and skeletal muscle mass), muscle architecture (muscle thickness, pennation angle, and fascicle length), questionnaires scores (i.e., GPAQ, FACT-G, and FACIT-F), mechanical parameters (i.e., critical force, *W*’, and total work done) and neuromuscular function parameters (i.e., MVC, RMS_%MVC_, Q_tw_, voluntary activation, M_max_, MEP.cM_max_^−1^) between groups. Two-way ANOVAs with repeated measures (group $\times$ time) were used to compare fatigue parameters. The time effect of the ANOVA included all data available of the parameter of interest pre, during and postexercise. Specifically, it included preexercise, MVC 1 to 60 and the recovery period (1, 2, 3, 5 and 10 min) for MVC and RMS_%MVC_; MVC 1, 10, 20, 30, 40, 50 and 60 and the recovery period for Q_tw_, voluntary activation, CT, HRT, RFD and M_max_; preexercise values and the recovery period for MEP.cM_max_^−1^. Multiple comparison analysis was performed with the Sidak post hoc test when a significant difference was found. Statistical significance was set at *P* < 0.05.

## Results

### Characteristics and baseline values of the two populations

Participants’ characteristics are reported in Table 1. Patients and healthy controls were matched for age, weigh, height, and physical activity level. Moreover, fat-free mass (*P* = 0.539), fat mass (*P* = 0.838) and skeletal muscle mass (*P* = 0.262) were not different between the two groups.

**Fig. 2:**
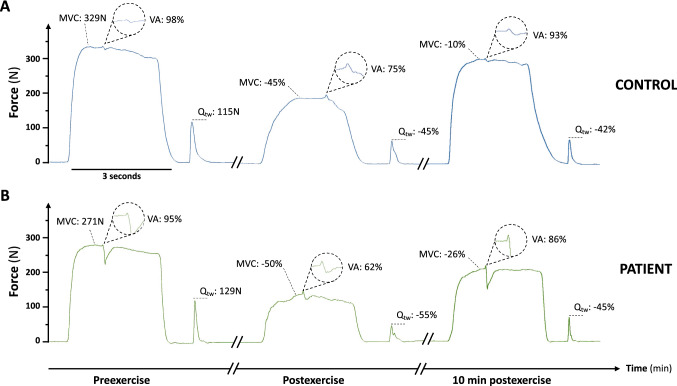
Representative traces of quadriceps maximal voluntary force (MVC) and quadriceps twitch force (Q_tw_) for a control (**A**) and the corresponding patient (**B**). *VA* voluntary activation. Significant time, group and interaction effects are indicated on the graphs for both exercise and recovery

Preexercise MVC was lower in patients than in controls (− 15 ± 18%, *P* = 0.022, Fig. 2). Preexercise VA and Q_tw_ were not different between groups (*P* = 0.589 and *P* = 0.523, respectively, Table 1) as well as Q_tw_ associated mechanics (CT, *P* = 0.782; HRT, *P* = 0.179; and RFD, *P* = 0.637; Table 2). There was also no difference between groups on RMS_%MVC_ for the three muscle groups (VL: *P* = 0.853; VM: *P* = 0.407; RF: *P* = 0.504). M-wave amplitudes were lower in patients than controls (Table 2). VL and RF MEP.cM_max_^−1^ were higher, and VM MEP.cM_max_^−1^ was similar in patients compared to controls (Table 2).

**Table 2:** Neuromuscular function of the quadriceps pre- and postexercise in patients and healthy controls

Parameters	Group	Preexercise	Exercise						
			MVC 1	MVC 10	MVC 20	MVC 30	MVC 40	MVC 50	MVC 60
CT, ms	Controls	103 ± 11	97 ± 11	104 ± 11	108 ± 7	105 ± 7	104 ± 8	103 ± 10	101 ± 10
	Patients	104 ± 11	97 ± 9	106 ± 12	113 ± 20	113 ± 15	113 ± 16	111 ± 16	109 ± 14
HRT, ms	Controls	77 ± 15	60 ± 15	76 ± 34	75 ± 30	72 ± 30	63 ± 21	64 ± 20	59 ± 19
	Patients	69 ± 18	56 ± 18	79 ± 54	79 ± 61	62 ± 29	72 ± 57	61 ± 38	55 ± 25
RFD, N.s^−1^	Controls	1393 ± 1242	3975 ± 1341	3368 ± 1317	2245 ± 1317	1920 ± 1383	1825 ± 1253	1665 ± 888	1831 ± 1139
	Patients	4207 ± 853	3364 ± 761	2728 ± 878	1603 ± 755	1419 ± 700	1403 ± 716	1380 ± 676	1387 ± 667
M_max_, mV									
VL	Controls	11.4 ± 2.9	11.0 ± 2.9	11.7 ± 3.0	11.8 ± 3.2	11.8 ± 3.3	11.7 ± 3.2	11.7 ± 3.1	11.7 ± 3.1
	Patients	7.7 ± 2.2	7.2 ± 2.1	7.9 ± 2.3	8.0 ± 2.3	8.0 ± 2.2	7.9 ± 2.2	8.0 ± 2.2	8.0 ± 2.2
VM	Controls	10.9 ± 3.8	10.5 ± 4.0	11.3 ± 4.5	10.9 ± 5.0	10.8 ± 4.9	10.4 ± 5.2	10.2 ± 4.7	10.4 ± 4.8
	Patients	6.7 ± 3.4	6.3 ± 3.2	6.7 ± 3.7	6.5 ± 3.6	6.3 ± 3.6	6.6 ± 3.6	6.6 ± 3.6	6.7 ± 3.5
RF	Controls	4.4 ± 1.5	4.4 ± 1.5	4.8 ± 1.8	4.7 ± 1.9	4.6 ± 1.9	4.7 ± 2.0	4.6 ± 1.9	4.8 ± 1.9
	Patients	3.2 ± 1.2	3.1 ± 1.1	3.5 ± 1.2	3.3 ± 1.2	3.3 ± 1.1	3.3 ± 1.1	3.3 ± 1.1	3.3 ± 1.1
MEP.cM_max_^−1^									
VL	Controls	0.18 ± 0.08	–	–	–	–	–	–	–
	Patients	0.25 ± 0.12	–	–	–	–	–	–	–
VM	Controls	0.20 ± 0.12	–	–	–	–	–	–	–
	Patients	0.25 ± 0.16	–	–	–	–	–	–	–
RF	Controls	0.27 ± 0.16	–	–	–	–	–	–	–
	Patients	0.39 ± 0.17	–	–	–	–	–	–	–

Parameters	Effects (*P*-value)			Recovery					Effects (*P*-value)		
	Time	Group	Interaction	1 Min	2 Min	3 Min	5 Min	10 Min	Time	Group	Interaction
CT, ms	0.006	0.152	0.133	99 ± 9	99 ± 9	99 ± 9	99 ± 9	99 ± 10	0.0441	0.139	0.069
				107 ± 14	105 ± 11	103 ± 11	103 ± 11	102 ± 11			
HRT, ms	0.016	0.902	0.581	67 ± 21	64 ± 19	57 ± 16	53 ± 13	47 ± 9	< 0.001	0.671	0.577
				57 ± 23	70 ± 58	56 ± 21	53 ± 21	44 ± 12			
RFD, N.s^−1^	< 0.001	0.166	0.734	2497 ± 1157	2577 ± 1116	2514 ± 1001	2401 ± 773	2275 ± 757	< 0.001	0.034	< 0.001
				2052 ± 771	2075 ± 754	2151 ± 658	2021 ± 616	2132 ± 562			
M_max_, mV											
VL	0.572	< 0.001	0.999	11.8 ± 3.3	11.8 ± 3.2	11.7 ± 3.1	11.3 ± 3.0	10.9 ± 3.0	< 0.001	< 0.001	0.348
				8.0 ± 2.2	8.0 ± 2.2	7.9 ± 2.1	7.7 ± 2.1	7.4 ± 2.1			
VM	0.464	0.008	0.776	10.9 ± 3.9	10.7 ± 3.9	10.9 ± 3.8	10.7 ± 3.6	10.5 ± 3.4	0.157	0.006	0.557
				6.8 ± 3.4	6.9 ± 3.4	7.0 ± 3.4	6.8 ± 3.3	6.7 ± 3.1			
RF	0.049	0.024	0.829	4.7 ± 1.7	4.7 ± 1.7	4.7 ± 1.8	4.6 ± 1.7	4.4 ± 1.6	< 0.001	0.014	0.305
				3.3 ± 1.0	3.3 ± 1.1	3.3 ± 1.1	3.2 ± 1.1	3.0 ± 1.1			
MEP.cM_max_^−1^											
VL	–	0.043	–	0.20 ± 0.14	0.21 ± 0.14	0.21 ± 0.14	0.24 ± 0.16	0.23 ± 0.13	0.035	0.128	0.656
	–		–	0.26 ± 0.10	0.28 ± 0.09	0.28 ± 0.17	0.28 ± 0.11	0.32 ± 0.16			
VM	–	0.283	–	0.25 ± 0.23	0.29 ± 0.32	0.24 ± 0.22	0.26 ± 0.23	0.26 ± 0.23	0.090	0.735	0.596
	–		–	0.25 ± 0.14	0.28 ± 0.13	0.27 ± 0.19	0.28 ± 0.15	0.31 ± 0.20			
RF	–	0.045	–	0.32 ± 0.19	0.31 ± 0.19	0.31 ± 0.19	0.34 ± 0.19	0.34 ± 0.20	0.035	0.070	0.936
	–		–	0.44 ± 0.19	0.45 ± 0.18	0.42 ± 0.20	0.44 ± 0.17	0.47 ± 0.20			

Data are presented as means ± SD

*CT* contraction time, *HRT* half-relaxation time, *MEP* motor evoked potential, *MEP.cM*_*max*_^*−1*^ MEP normalized to concomitant M_max_, *M*_*max*_ maximal M-wave, *RF* rectus femoris, *RFD* maximal rate of force development, *VL* vastus lateralis, *VM* vastus medialis

**Fig. 3:**
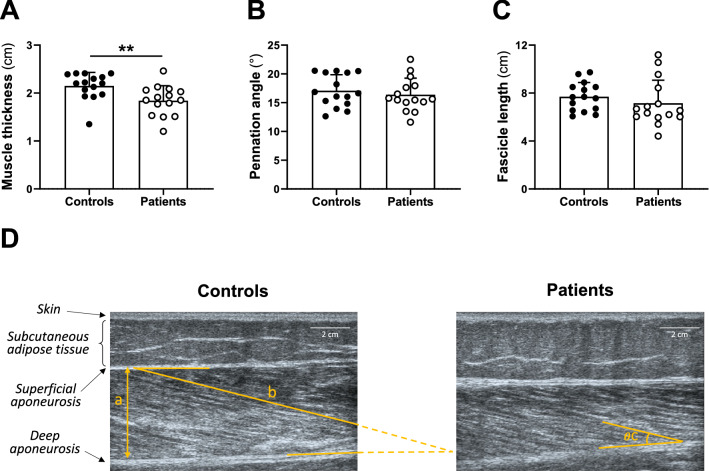
Muscle architecture of the vastus lateralis investigated by ultrasonography. Muscle thickness (**A**), fascicle length (**B**), and pennation angle (**C**) were measured in controls (black circles) and patients (white circles). Example of ultrasound images of the vastus lateralis in a representative patient and her matched healthy control (**D**) showing analyses of muscle architecture (a, muscle thickness; b, fascicle length; c, pennation angle). Data are presented as the means ± SD. Patients are presented in white circles (*n* = 15), and controls are presented in black circles (*n* = 15). **Significant difference between groups (*P* < 0.01)

As illustrated in Fig. 3, muscle thickness was lower in patients than controls (− 13.4 ± 14.8%, *P* = 0.004, Fig. 3A), while no difference was observed between groups for pennation angle (*P* = 0.515, Fig. 3B) and fascicle length (*P* = 0.362, Fig. 3C) in the vastus lateralis muscle.

Scores of cancer-related fatigue and quality of life are reported in Table 3. The FACIT-F score was significantly lower in patients than in controls (*P* < 0.001). Each item for quality of life was lower in patients than in controls (all *P* < 0.01), except for social well-being.

**Table 3:** Quality of life and perceived fatigue in patients and healthy controls

	Controls (*n* = 15)	Patients (*n* = 15)	*P* value
FACT-G	101 ± 12	78 ± 12	< 0.001
Physical well-being	32 ± 1	19 ± 6	0.001
Social well-being	22 ± 5	22 ± 6	0.660
Emotional well-being	26 ± 5	20 ± 3	0.001
Functional well-being	21 ± 5	17 ± 3	0.007
FACIT-F	47 ± 4	32 ± 11	< 0.001
Total score	148 ± 15	110 ± 21	< 0.001

Data are presented as means ± SD. *FACT-G* functional assessment of cancer therapy—general, *FACIT-F* functional assessment of chronic illness therapy—fatigue

### 60-MVC protocol

Preexercise MVC values did not differ from the first MVC of the exercise (patients: 303 ± 54 N vs. 297 ± 56 N, *P* = 0.732; controls: 368 ± 89 N vs. 365 ± 87 N, *P* = 0.919), indicating no pacing strategy by the participants at the start of the 60-MVC protocol.

#### Parameters associated with the force-duration relationship

**Fig. 4:**
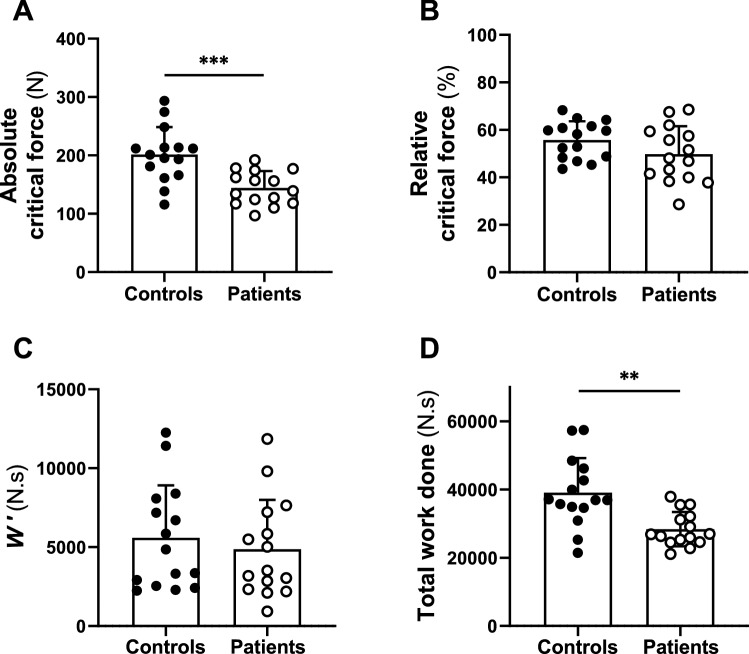
Parameters of the force-duration relationship. Critical force is expressed in absolute units (**A**) and normalized as a percent of preexercise (**B**). *W*’ is the work performed above critical force (**C**) and total work done (**D**) is the total work of the task. Data are presented as the means ± SD. Patients are presented in white circles (*n* = 15), and controls are presented in black circles (*n* = 15). **Significant difference between groups (*P* < 0.01); ***Significant difference between groups (*P* < 0.001)

As displayed in Fig. 4A, the absolute critical force was 24 ± 24% lower in patients than in controls (144 ± 29 N vs. 201 ± 47 N, respectively, *P* < 0.001) and represented 50 ± 12% and 56 ± 8% of the MVC, respectively (*P* = 0.116, Fig. 4B). As illustrated in Fig. 4C, *W*’ was not different between groups (patients: 4869 ± 3122 N.s; controls: 5590 ± 3331 N.s, *P* = 0.546). As shown in Fig. 4D, total work done was 23 ± 22% lower in patients than in controls (25,972 ± 5199 U.s^−1^ vs. 36,244 ± 8497 U.s^−1^, respectively, *P* = 0.001).

#### Force

**Fig. 5:**
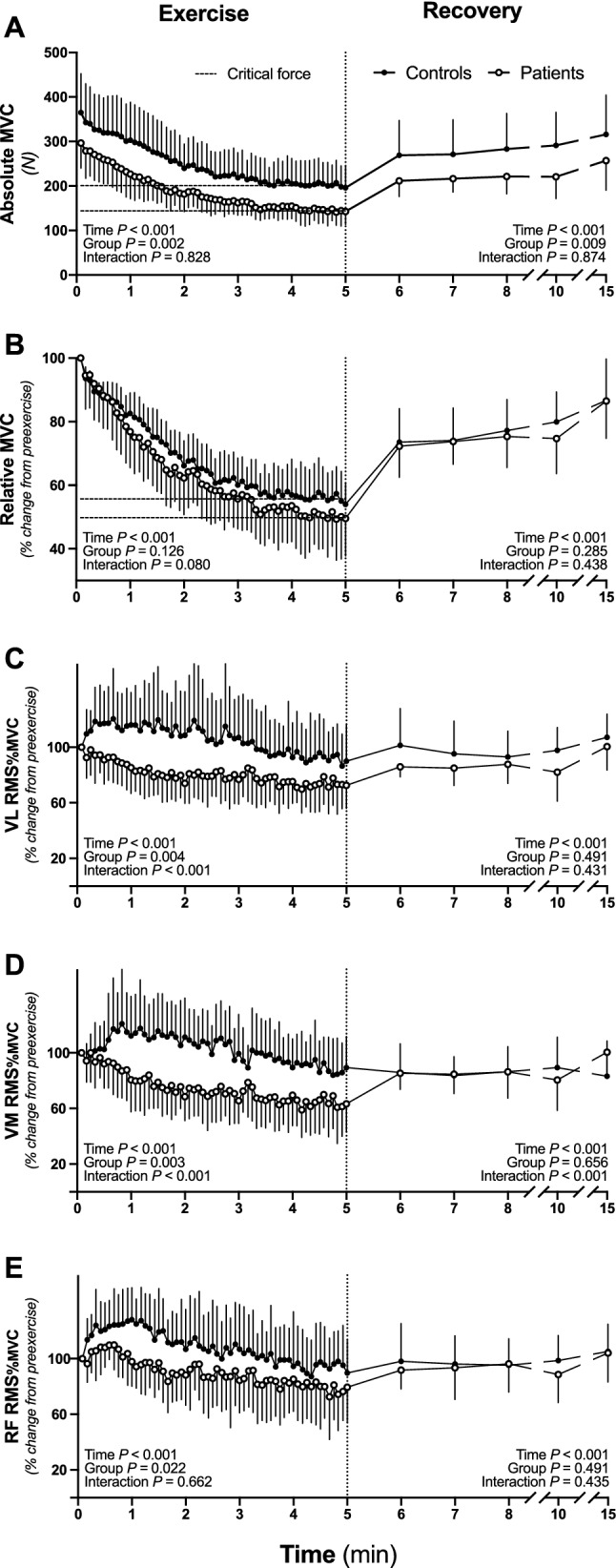
Quadriceps maximal voluntary force (MVC) and muscle activation during the 60-MVC protocol and during recovery. MVC is expressed in absolute units (**A**) and as a percentage of preexercise (**B**). Vastus lateralis (**C**), vastus medialis (**D**) and rectus femoris (**E**) muscle activation are expressed as a percentage from preexercise. Data are presented as the means ± SD. Patients are presented in white circles (*n* = 15), and controls are presented in black circles (*n* = 15). Dotted lines display critical force

The profile of quadriceps force output during the 60 MVCs and the subsequent 10 min recovery period is shown in Fig. 5. When expressed as absolute values (Fig. 5A), a significant group effect was found, indicating lower MVCs in patients than in controls throughout the exercise (− 21 ± 24%, *P* = 0.002). No interaction (group $\times$ time) effect was found, indicating that MVCs were shifted downward in patients compared to controls. When MVC data were normalized to preexercise (Fig. 5B), no group or interaction (group $\times$ time) effects were observed, indicating that patients and controls displayed similar neuromuscular fatigue development (ΔMVCs pre- to postexercise: − 51.6 ± 11.1% vs. − 46.4 ± 7.5%, respectively, *P* = 0.126). During exercise, quadriceps force decreased (*P* < 0.001) from the first to the 38th MVC in controls, and from the first to the 40th MVC in patients. Thereafter, quadriceps force plateaued until the last MVC for both groups. Similar to the results during exercise, no group or interaction effect was observed for MVCs during recovery when data were normalized to preexercise (Fig. 5B), indicating similar recovery capacity between patients and controls. However, while absolute MVCs were lower throughout exercise, they remained lower during recovery in patients than in controls (Fig. 5A). In both groups, MVC remained lower 10 min postexercise compared to preexercise (patients: − 13.6 ± 11.8%,* P* = 0.003; controls: − 15.6 ± 12.1%, *P* = 0.018) (Fig. 5B).

#### Electromyography

Changes in VL, VM, and RF RMS_%MVC_ during exercise are shown in Fig. 5. Similar to the force output, RMS_%MVC_ decreased during the exercise protocol in both groups (*P* < 0.001). A significant group effect was found, indicating lower muscle activation in patients than controls throughout the exercise for VL (Fig. 5C), VM (Fig. 5D), and RF (Fig. 5E) RMS_%MVC_. During recovery, no between-group differences were found in RMS_%MVC_. Pre- and postexercise VL, VM, and RF M_max_ amplitudes are shown in Table 2. During the 60-MVC protocol, no alteration in VL and VM M_max_ amplitudes was observed, while RF M_max_ amplitude increased (*P* = 0.049). There was a group effect for the three muscle groups (VL: *P* < 0.001; VM: *P* < 0.008; RF: *P* = 0.024), indicating that M_max_ amplitudes remained lower throughout exercise in patients than in controls (Table 2).

#### Central fatigue

**Fig. 6:**
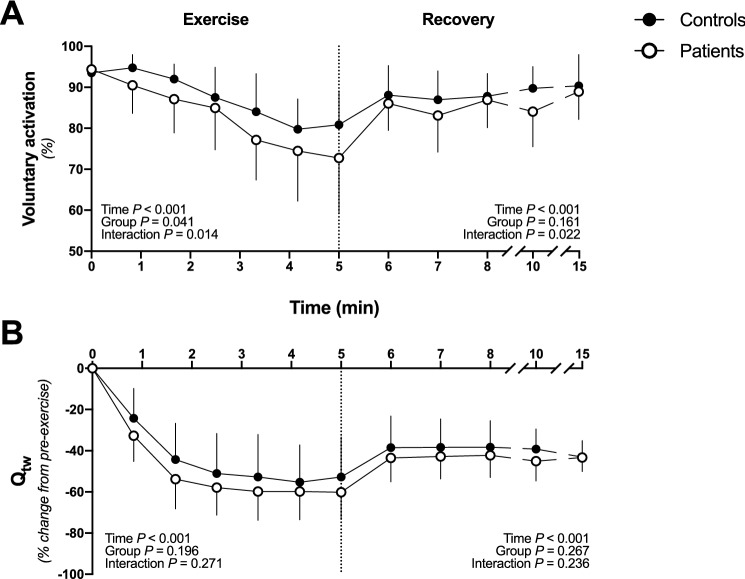
The development of peripheral and central fatigue during the 60-MVC protocol and during recovery. **A** and **B** show changes in voluntary activation and Q_tw_ throughout exercise and recovery, respectively. Data are presented as the means ± SD. Patients are presented in white circles (*n* = 15), and controls are presented in black circles (*n* = 15). Significant time, group and interaction effects are indicated on the graphs for both exercise and recovery. *Q*_*tw*_ quadriceps twitch force

During exercise, voluntary activation significantly decreased over time in both groups (*P* < 0.001). Moreover, significant group and interaction (group $\times$ time) effects were found for voluntary activation (Fig. 6A). Specifically, the decrease in voluntary activation was greater in patients than in controls (ΔVA pre- to postexercise: − 21.6 ± 13.3% vs. − 12.6 ± 7.7%, respectively, *P* = 0.041), indicating exacerbated central fatigue in patients. During recovery, a significant interaction (group $\times$ time) effect was found, indicating different recovery kinetics in voluntary activation between patients and controls (Fig. 6A). In particular, voluntary activation returned to preexercise levels after 10 min of recovery in controls (− 3.2 ± 4.5%, *P* = 0.122), while it did not return to preexercise values after 10 min of recovery in patients (− 5.4 ± 5.6%, *P* = 0.029). VL, VM, and RF MEP.cM_max_^−1^ increased following exercise in both groups, with no group or interaction effect, indicating no difference in corticospinal excitability between patients and controls (Table 2).

#### Peripheral fatigue

During exercise, Q_tw_ significantly decreased over time (*P* < 0.001), but there was no group or interaction (i.e., group $\times$ time) effect found (Fig. 6B), indicating a similar level of peripheral fatigue between patients and controls (ΔQ_tw_ pre- to postexercise: − 60.2 ± 13.2% vs. − 52.8 ± 19.4%, respectively, *P* = 0.196). That said, Q_tw_ gradually decreased from the first to the 20th MVC in patients and from the first to the 30th MVC in controls. Thereafter, Q_tw_ plateaued for both groups until the last MVC of the trial. Similarly, the contractile properties of Q_tw_ (i.e., CT, HRT, and RFD) decreased during exercise, with no difference between patients and controls (Table 2). Similar to MVCs, Q_tw_ recovery was not different between groups and remained lower compared to preexercise 10 min postexercise (patients: − 43.3 ± 6.8%, *P* < 0.001; controls: − 43.1 ± 8.0%, *P* < 0.001) (Fig. 6B).

## Discussion

The present study aimed to characterize the etiology of exercise-induced neuromuscular fatigue and its consequences on the force-duration relationship to provide new mechanistic insights into the reduced exercise capacity characterizing patients with early-stage breast cancer at chemotherapy completion. Compared to healthy controls matched for age, weight, height, and physical activity level, patients displayed a ~ 23% reduction in total work done at chemotherapy completion as a consequence of a lower maximal force and absolute critical force but similar *W*’. Importantly, this substantial impairment in exercise capacity was associated with exacerbated central fatigue during exercise in patients compared to their healthy counterparts, while peripheral fatigue was similar. Therefore, the current findings support central fatigue as a primary cause of the reduction in exercise capacity characterizing early-stage breast cancer patients treated with chemotherapy.

### Exercise capacity in breast cancer patients

Breast cancer patients showed a substantial reduction in exercise capacity at chemotherapy completion compared to their healthy counterparts. In this context, the 60-MVC exercise protocol was selected to provide a cohesive framework within which to investigate the mechanistic bases of this impairment in exercise capacity. Indeed, the reduced exercise capacity observed in early breast cancer patients, evidenced by a ~ 23% reduction in total work done, was characterized by lower maximal force and absolute critical force but similar *W*’. In other words, we observed a downward shift in the MVC curve in patients compared to controls (Fig. 5A). As a consequence, when expressed relative to maximal force (i.e., preexercise MVC), the critical force was similar in patients and controls, indicating that early breast cancer led to a proportional reduction in exercise capacity in the severe and the heavy intensity domains (Poole et al. 2016). However, we also documented a ~ 24% reduction in absolute critical force in patients compared to controls (Fig. 4A), which highlights the magnitude of the reduction of the greatest metabolic rate that results in wholly oxidative energy provision (Poole et al. 2016). In contrast, we found a similar magnitude of *W*’ between groups, a parameter that has been associated with the development of neuromuscular fatigue (Poole et al. 2016; Zarzissi et al. 2020) and the loss of muscular efficiency (Murgatroyd and Wylde 2011). This result is in contradiction with our hypothesis (i.e., greater *W*’ in patients than controls) but consistent with the observation of similar, instead of greater, level of neuromuscular fatigue between patients and their healthy counterparts. Therefore, these results provide novel insights from a bioenergetics perspective into the reduction in exercise capacity characterizing patients with early breast cancer.

### Exacerbated central fatigue in breast cancer patients

The reduction in exercise capacity observed in breast cancer patients was associated with an exacerbated central fatigue, as evidenced by the reduction in voluntary activation measured immediately at exercise cessation, compared to their healthy counterparts (Fig. 6A). This result is supported by previous research suggesting, through deductive inferences (i.e., central fatigue was not directly measured), that the mechanisms related to exacerbated fatigue originated within the central nervous system in cancer survivors with solid tumors (Cai et al. 2014; Kisiel-Sajewicz et al. 2012, 2013; Yavuzsen et al. 2009). Mechanistically, greater central fatigue was likely not explained in the present study by reduced corticospinal excitability, as evidenced by the absence of difference between groups (Table 2). However, it is important to note that several studies documented that corticospinal excitability remained unaltered in the face of a decrease in voluntary activation following fatiguing exercise (Gandevia et al. 1996; Kalmar and Cafarelli 2006). Therefore, the lack of a net effect on corticospinal excitability does not necessarily designate the absence of change but a counterbalance of excitatory and inhibitory influences on the corticospinal pathway (Weavil and Amann 2018).

Importantly, greater central fatigue was observed in patients, while peripheral fatigue was similar compared to controls. Taken together, and given the tight relationship between intramuscular metabolic perturbation and peripheral fatigue (Hureau et al. 2022), these results might suggest overactive group III/IV muscle afferent feedback in patients with breast cancer, a phenomena that have already been evidenced in other chronic diseases (Gagnon et al. 2012; Amann et al. 2014). Indeed, metabo- and mechanosensitive group III/IV sensory neurons project from skeletal muscles to the central nervous system and promote central fatigue during intense exercise to restrict peripheral fatigue and protect the exercising muscles from severe threats to muscle homeostasis (Blain et al. 2016; Hureau et al. 2018). Further studies specifically investigating group III/IV muscle afferent feedback in patients with breast cancer are needed to evaluate whether this mechanism is involved in the central maladaptations observed in the present study.

### Peripheral fatigue in breast cancer patients

The present study demonstrated that peripheral fatigue, as evidenced by the decrease in Q_tw_, was similar in breast cancer patients compared to healthy controls (Fig. 6B). Based on this result, peripheral fatigue seems relatively preserved during chemotherapy. Interestingly, a recent study investigated the determinants of cancer-related fatigue by separating fatigued and nonfatigued cancer survivors into two groups based on a clinical cutoff point (Brownstein et al. 2022). This study documented that clinically fatigued patients experienced more peripheral fatigue during cycling exercise than those reporting no clinical fatigue. In contrast with the present study, this investigation demonstrates the existence of peripheral alterations in breast cancer survivors. This discrepancy between studies might be explained by the difference in the populations investigated (i.e., fatigued and nonfatigued patients vs. patients and healthy controls) and/or the exercise modality utilized to induce neuromuscular fatigue (i.e., whole-body cycling vs. single-leg extension).

Regardless, the observation of similar *acute* peripheral fatigue between patients and controls does not rule out the presence of *chronic* peripheral alterations subsequent to chemotherapy. Indeed, patients displayed a ~ 15% reduction in MVC at baseline compared to their healthy counterparts. Moreover, our results showed reduced skeletal muscle mass, as evidenced by lower vastus lateralis muscle thickness measured via ultrasonography, which is supported by a previous study showing a reduced muscle cross-sectional area in breast cancer patients compared to healthy controls via vastus lateralis muscle biopsies (Guigni et al. 2018b). In addition to this lower muscle quantity, impairments in muscle quality have been previously identified in breast cancer patients, such as a reduction in mitochondrial content (Mallard et al. 2022c). Therefore, profound chronic changes in both muscle quantity and quality are likely responsible for the reduction in MVC observed in patients compared to controls.

### Methodological considerations

The present study investigated the etiology of neuromuscular fatigue in an homogeneous group of patients (i.e., from one cancer type) compared to healthy controls considering the large heterogeneity of symptoms, treatment options, and outcomes across cancers (Lin 2019; Hickok et al. 2005; Baracos et al. 2018). While the control group was matched one-to-one with a patient for age, weight, height, and physical activity level, patients displayed greater subcutaneous adipose tissue on the vastus lateralis muscle than controls, which might affect EMG recording (Farina et al. 2004). However, EMG data were normalized (RMS_%MVC_) to allow relative comparisons between groups. In addition to MEPs, analyses of the silent period resulting from transcranial magnetic stimulations could have provided more information on intracortical inhibition. However, this study was not designed to measure the silent period as we did not give participants the specific instructions that are required to assess it rigorously (Hupfeld et al. 2020).

## Conclusion

The present study provides new mechanistic insights into the etiology of exercise-induced neuromuscular fatigue and its consequences on the force-duration relationship in early-stage breast cancer patients at chemotherapy completion. Patients demonstrated a substantial reduction in exercise capacity, evidenced by a lower total work done, and characterized by a lower maximal force and absolute critical force but similar *W*’. Importantly, this impaired exercise capacity was associated with exacerbated central fatigue during exercise in patients compared to their healthy counterparts, while peripheral fatigue was similar. Therefore, the current findings support central fatigue as a primary cause of the reduced exercise capacity characterizing early-stage breast cancer patients treated with chemotherapy. Further studies are needed to elucidate the specific mechanisms responsible for the development of this exacerbated level of central fatigue. Moreover, studies focusing on supportive care to counteract this maladaptation are also of interest to alleviate, at least in part, cancer-related fatigue, the most common symptom in cancer patients.

## Data Availability

The datasets generated during and/or analysed during the current study are available from the corresponding author on reasonable request.

## Abbreviations

ANOVA: Analysis of variance
BF: Biceps femoris
CT: Contraction time
EMG: Electromyography
HRT: Half relaxation time
MEP: Motor evoked potentials
M_max_: Maximal M-wave
RFD: Maximal rate of force development
MVC: Maximal voluntary contraction
Q_tw_: Maximal single twitch peak force
RF: Rectus femoris
RMS: Root mean square
VL: Vastus lateralis
VM: Vastus medialis
